# The *rnb* Gene of *Synechocystis* PCC6803 Encodes a RNA Hydrolase Displaying RNase II and Not RNase R Enzymatic Properties

**DOI:** 10.1371/journal.pone.0032690

**Published:** 2012-03-05

**Authors:** Rute G. Matos, Arsénio M. Fialho, Mordechai Giloh, Gadi Schuster, Cecília M. Arraiano

**Affiliations:** 1 Instituto de Tecnologia Química e Biológica/Universidade Nova de Lisboa, Oeiras, Portugal; 2 Institute for Biotechnology and Bioengineering (IBB), Centre for Biological and Chemical Engineering/Instituto Superior Técnico, Lisboa, Portugal; 3 Faculty of Biology, Technion-Israel Institute of Technology, Haifa, Israel; Keio University, Japan

## Abstract

Cyanobacteria are photosynthetic prokaryotic organisms that share characteristics with bacteria and chloroplasts regarding mRNA degradation. *Synechocystis sp.* PCC6803 is a model organism for cyanobacteria, but not much is known about the mechanism of RNA degradation. Only one member of the RNase II-family is present in the genome of *Synechocystis sp* PCC6803. This protein was shown to be essential for its viability, which indicates that it may have a crucial role in the metabolism of *Synechocystis* RNA. The aim of this work was to characterize the activity of the RNase II/R homologue present in *Synechocystis sp.* PCC6803. The results showed that as expected, it displayed hydrolytic activity and released nucleoside monophosphates. When compared to two *E. coli* counterparts, the activity assays showed that the *Synechocystis* protein displays RNase II, and not RNase R characteristics. This is the first reported case where when only one member of the RNase II/R family exists it displays RNase II and not RNase R characteristics.

## Introduction

Cyanobacteria are photosynthetic prokaryotic organisms comprising the major biomass of living organisms in earth oceans, and are believed to be related to the ancestor of higher plant chloroplasts. They share characteristics from bacteria and chloroplasts regarding mRNA degradation. *Synechocystis* PCC6803 is a model organism for cyanobacteria, but not much is known about the mechanism of RNA degradation in this class of organisms.

The *Synechocystis* genome contains genes that have a high homology to RNase E, RNase J, PNPase, RNase II/R and nucleotidyltransferase/poly(A)-polymerase (PAP), the proteins involved in mRNA degradation [1]. The polyadenylation pathway was already studied and it was shown that the product of the putative PAP gene has nucleotidyltransferase and not polyadenylation activity. Instead, the reaction of polyadenylation in *Synechocystis* is performed by PNPase, which generates heterogeneous poly(A)-rich tails [1]. In the chloroplast of higher plants, PAP together with PNPase contributes to the polyadenylation activity [2], [3]. In *Synechocystis* the absence of PNPase is lethal for the cell [1]. The same behaviour was observed by disrupting the genes for RNase II/R or RNase E [1]. In *Synechocystis*, the RNase E homologue is more related to RNase G and it is not part of a multicomponent complex, unlike RNase E of *Escherichia coli* [1]. However, *in vivo* assays showed that RNase E homologue from *Synechocystis* is able to complement the functions of both RNase E and RNase G in *E. coli*. Moreover, its endonucleolytic activity was confirmed *in vitro*, showing that cleavage is dependent on the primary target sequence and on the secondary structure of the mRNA [4]. In addition to RNase E, the *Synechocystis* genome contains an RNase J homologue, a ribonuclease that has both endonucleolytic and 5′ to 3′ exonucleolytic activity and was originally described in *Bacillus subtilis* [5], [6].

Analysis of the *Synechocystis sp*. genome has revealed a single member of the RNase II-family of proteins, from now on named synRNB. Proteins from this family are present in all domains of life, are involved in several processes, and play a central role in the mechanism of gene expression [7]. In eukaryotes, RNase II homologues are part of a multiprotein complex, called the exosome, where they provide the catalytic subunit [8]. Mutants in these homologues have shown defects in development, mitotic control and chloroplast biogenesis [9]. In prokaryotes, they are important for growth and stress responses and they are also involved in virulence [7], [9]. RNase II is the prototype of this family of enzymes, which also comprises RNase R in *E. coli*. They both act hydrolytically, degrading RNA molecules in the 3′ to 5′ direction, releasing 5′-nucleoside monophosphates [10]. The resolution of RNase II crystal structure showed that it is composed of two N-terminal Cold Shock Domains (CSD) domains and a C-terminal S1 domain involved in RNA binding [11], [12]. The central RNB domain is responsible for the catalytic activity of the protein [11]–[13]. This domain organization is present in all family members [11], [14]. In the RNase II catalytic cavity, the first five nucleotides of the RNA molecule (counting from the 3′ end) are stacked between the two aromatic residues, Tyr253 and Phe358. After the last cleavage event, the 4 nt fragment is no longer “clamped” by those residues, and then the 4 nt is the end-product released by RNase II [11]. Mutational analysis showed that the Tyr253 is crucial for setting the final end product, not only in RNase II [15] but also in RNase R [13], indicating that its role may be conserved in all members of this family of enzymes. The active site is formed by four highly conserved aspartates (Asp201, Asp207, Asp209 and Asp210) and an arginine (Arg500), which were shown to be important for the activity of the protein and proper RNA binding/orientation [15]–[17]. From these residues, Asp209 was the most critical for the activity of RNase II, since its substitution for an asparagine totally abolished its activity without affecting the RNA binding ability [16]. The same results were obtained for other members of this family [8], [13], [18], [19]. It was postulated that Glu542 was involved in nucleotide elimination. However, when Glu542 was changed into an alanine, the resultant protein had more affinity for RNA and it was more than 100 times more active than the wild type protein. Therefore, the E542A mutant was called a “super-enzyme” [17].

*E. coli* RNase II and RNase R belong to the same family, but act differently on RNA substrates and have different specificities. RNase R was shown to have an intrinsic ability to unwind double-stranded RNAs [18], [20], while RNase II is sensitive to these structures and stalls a few nucleotides before reaching the double-stranded region [7]. RNase II is responsible for 90% of the hydrolytic activity in *E. coli* [21]; however, in stationary phase and stress conditions (like cold-shock induction), RNase R levels increase in the cell [22]. This can be related to the crucial role that RNase R has in RNA quality control, namely in the degradation of defective tRNA and rRNA [23], and also in protein quality control [22]. In contrast, RNase II is mainly involved in the terminal stages of mRNA degradation and its activity can be replaced by PNPase. It was also suggested that the main function of RNase II is the protection of some mRNA transcripts by rapidly removing the short poly(A) tail [24].

In cyanobacteria nothing is known about this family of enzymes, except that SynRNB protein is essential for the viability of these organisms [1]. This indicates that RNase II/R homologue may have a crucial role in *Synechocystis* metabolism. The aim of this work was to characterize the activity of synRNB as a first approach for the study of the role of this enzyme in RNA metabolism in cyanobacteria. The results showed that the *Synechocystis* protein behaves like an RNase II-like protein: the final product released is a 4 nt fragment and the protein is not able to degrade structured substrates. This is the first case reported where the only member of the RNase II family of enzymes present in an organism behaves like an RNase II and not like RNase R. However, while RNase II prefers polyadenylated substrates [15], [17], RNase II from *Synechocystis* does not demonstrate such preference. This may happen because in this genus there is no PAP and the tails produced by PNPase are heterogeneous [1].

## Results and Discussion

### RNB protein from *Synechocystis sp.* PCC6803 is a hydrolytic exoribonuclease

**Figure 1:**
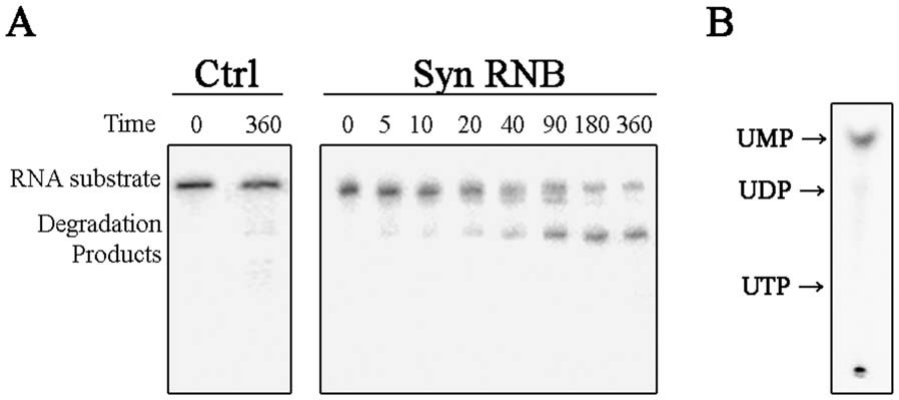
RNA degradation activity of synRNB. **A.** 33.3 ng/µl of radiolabelled *petD3* RNA was incubated with 10 nM of protein at 37°C. Samples were taken during the reaction at the time points indicated in the figure. A control reaction with no enzyme added (Ctrl) was incubated at the maximum reaction time. **B.** TLC (Thin Layer Chromatography) analysis of the degradation products of *Synechocystis* recombinant protein. The RNA degradation products were chromatographed on a TLC screen. Unlabeled standards were loaded on the same plate and visualized with UV-light and their position is indicated by arrows.

SynRNB, purified by immobilised metal affinity chromatography, was incubated with petD3 RNA to test the activity of the protein after purification. After only 5 minutes of incubation we were already able to see the formation of degradation products, which increased in abundance with time (Figure 1A). To confirm that this activity was due to RNB from *Synechocystis* and did not result from a contamination with the *E. coli* PNPase, we analysed the reaction products by TLC (Thin Layer Chromatography) (Figure 1B) [25]. As shown in figure 1B, the products detected were exclusively nucleotide monophosphates. In this case, since the RNA was [^32^P] UTP labelled, the obtained radioactive product co-migrated with the UMP marker. With the results obtained we were able to confirm that the recombinant RNB from *Synechocystis* has hydrolytic activity.

### Salt and pH preference of the synRNB protein

In order to characterize the activity of synRNB protein we first determined the optimal conditions for the catalysis. We empirically determined the effects of changing the salt, its concentration and the pH of the reaction buffer.

**Figure 2:**
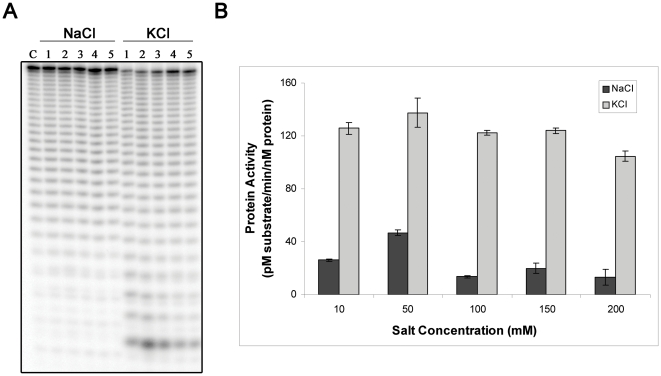
Salt dependence of synRNB. **A.** 5 nM of recombinant protein were incubated with 10 nM of Poly(A) at 37°C for 5 minutes in a reaction buffer with different salt concentrations (lanes 1 = 10 mM, lanes 2 = 50 mM, lanes 3 = 100 mM, lanes 4 = 150 mM, and lanes 5 = 200 mM). **B.** Determination of the protein activity in the presence of NaCl and KCl in different concentrations.

To assess the effect of salt type and concentration, we performed activity assays with 10, 50, 100, 150 and 200 mM of NaCl and KCl as described in the Materials and Methods. The activity of synRNB was measured by quantifying the disappearance of the substrate with time. The conditions used for this and the following determinations were adjusted such that less than 25% of the substrate was degraded. The results obtained are presented in figure 2. It should be noted that the gels showed in this figure and the ones that follow were not used to quantify the degradation of the substrate. In the ones that are shown, the reactions have been allowed to proceed further to enable detection of the final products. The assays quantified were performed in different conditions (lower protein concentration and reaction time) to ensure that less than 25% of the substrate was degraded. When the salt used was NaCl, the protein seemed inactive for all the concentrations tested, while in the presence of KCl we were able to detect activity with all the concentrations used, although the proteins preferred 50 mM KCl (Figure 2A). However, following the reactions ate different time points, we were able to see that, in fact, synRNB was active in NaCl, but the cleavage efficiency was 10 times lower when compared to its activity in KCl (Figure 2B). We were also able to confirm that the preferred KCl concentration was 50 mM. For higher salt concentrations, the activity of synRNB starts to decrease (Figure 2B). Taking these results into consideration, we used an activity buffer with a salt concentration of 50 mM of KCl for the remaining experiments.

**Figure 3:**
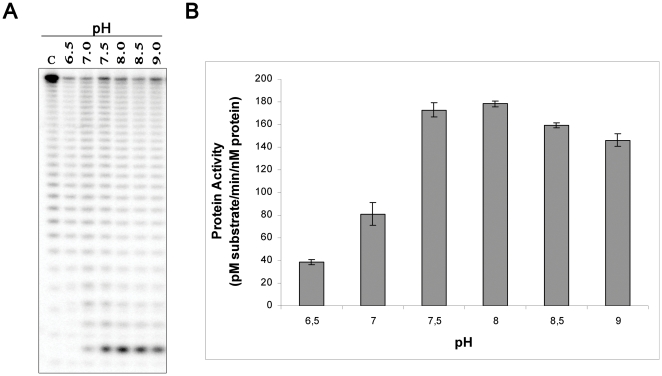
pH dependence of synRNB. **A.** 5 nM of recombinant protein were incubated with 10 nM of Poly(A) at 37°C for 5 minutes in a reaction buffer with different pH, ranging from 6.5 to 9. **B.** Determination of the protein activity in the presence of different pH.

After establishing the optimal salt concentration for the activity of the protein, we analysed the effect of pH on catalysis. For this purpose, we tested the activity of synRNB using pH ranging from 6.5 to 9 in the presence of 50 mM of KCl. The results obtained showed that the activity of synRNB peaked at a pH of 8.0 (Figure 3). However, the peak is relatively broad and the activity at pH 7.5 and 8.5 was not substantially lower (Figure 3B).

### RNB protein from *Synechocystis sp.* PCC6803 prefers Mg^2+^ for its activity

**Figure 4:**
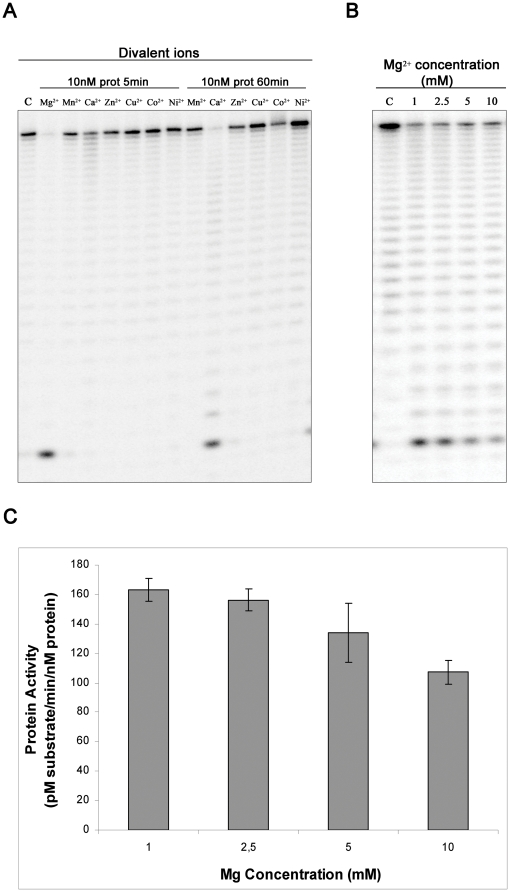
Divalent metal ion dependence of synRNB. **A.** 10 nM of recombinant protein were incubated with 10 nM of Poly(A) at 37°C for 5 and 60 minutes in a reaction buffer with different divalent metal ions. **B.** **A.** 5 nM of recombinant protein were incubated with 10 nM of Poly(A) at 37°C for 5 minutes in a reaction buffer with different Mg^2+^concentrations.**C.** Determination of the protein activity in the presence of different Mg^2+^ concentrations.

Exoribonucleases from the RNase II-family of enzymes need a divalent ion in order to proceed with catalysis. For *E. coli* RNase II and RNase R, the presence of Mg^2+^ is crucial for the activity of the proteins [11], however, catalysis can also occur in the presence of other divalent ions (Matos et al, manuscript submitted). We tested the activity of the *Synechocystis* homologue with different divalent metal ions: Mg^2+^, Mn^2+^, Ca^2+^, Zn^2+^, Cu^2+^, Co^2+^, and Ni^2+^. As shown in figure 4A, incubating the RNA with 10 nM of protein during 5 minutes we can only detect strong activity in the presence of Mg^2+^. In the presence of Ca^2+^, it seems that the protein has some residual activity. To confirm this, we increased the time of the reaction, and saw that, after one hour of incubation in a buffer with Ca^2+^, the protein was also able to cleave the substrate (Figure 4A). Under the same conditions no activity was detected for any of the other divalent ions that were tested (Figure 4A). From these experiments we conclude that the synRNB needs an Mg^2+^ ion for the catalysis, but is also able to cleave RNA in the presence of Ca^2+^, although with less efficiency. We then tested the effect of changing the Mg^2+^ concentrations (1, 2.5, 5 and 10 mM). As shown in figure 4B and C, we found that synRNB is most active in the presence of 1 mM of Mg^2+^. At higher Mg^2+^ concentrations, the activity of synRNB decreases (Figure 4B and C).

For the following experiments, we used reaction conditions that were optimised as described above; 50 mM KCl and 1 mM MgCl_2_ in 20 mM Tris-HCl buffer with a pH of 8.0. To prevent degradation of substrates during SPR experiments, Mg^2+^ was omitted and EDTA added to the buffer.

### RNB protein from *Synechocystis sp.* PCC6803 displays RNase II-like properties

**Figure 5:**
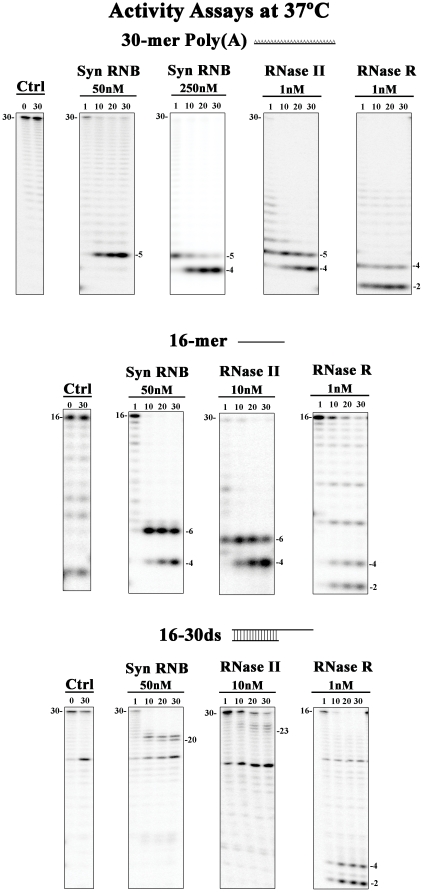
Exoribonucleolytic activity at 37°C of *Synechocystis* protein: comparison with *E. coli* RNase II and RNase R. Activity assays were performed using the three synthetic substrates: 30-mer poly(A), the 16-mer and the double-stranded substrate 16–30 ds. The concentration of proteins used is indicated in the figure. Samples were taken during the reaction at the time points indicated. Control reactions with no enzyme added (*Ctrl*) were incubated at the maximum reaction time for each protein. Length of substrates and degradation products are labelled.

RNase II and RNase R of *E. coli* differ with regard to the final product released and their ability to degrade double-stranded RNAs [13], [17]. While the *E. coli* RNase II releases an end-product of 4 nt and is sensitive to secondary structures, stalling 5 to 7 nt before it reaches the double-stranded region, RNase R degrades RNA releasing fragments of 2 nt of length and is able to overcome structured RNAs [13], [17]. We tested how the *Synechocystis* protein behaved regarding the degradation of two single-stranded substrates, poly(A) and 16 ss, and also using the double-stranded substrate 16–30 ds (Figure 5). In order to compare synRNB with *E. coli* RNase II and RNase R, the three proteins were assayed at the same time. The activity of RNase II and RNase R was assayed using the conditions described previously [16], [26], [27]. For the *Synechocystis* protein, the conditions used were those described above. The results obtained are represented in Figure 5. Different conditions were used for the three proteins (salt and/or Mg^2+^ concentrations) to ensure that all were acting in their optimal conditions.

It was already shown that *E. coli* RNase II and RNase R prefer poly(A) substrates [13], [17]. For that reason, this was one of the substrates that we used to test the activity of the *Synechocystis* protein from this family. We also decided to use the 16-mer RNA, which had a mixed composition of all ribonucleotides, in order to analyse if, like its *E. coli* homologues, synRNB had some preference for poly(A) substrates. As already described, and as observed for both single-stranded substrates tested in this work, RNase II is able to degrade ssRNA substrates releasing a 4 nt fragment, while RNase R is able to proceed with catalysis until it reaches the 2 nt of length (Figure 5) [7]. When we tested the recombinant protein from *Synechocystis* with the poly(A) substrate, we were able to see that, with 50 nM of protein, the final product released was a 5 nt fragment (Figure 5). However, when we used a higher protein concentration, 250 nM, the protein was now able to release a 4 nt end-product (Figure 5). We also assayed this substrate with higher protein concentrations but no more cleavage events were observed (data not shown), confirming that the final product released is 4 nt, as produced by *E. coli* RNase II. However, 50 nM of the *Synechocystis* protein were sufficient to degrade the 16 ss substrate until the 4 nt of length. Together, these results show that by leaving a 4 nt degradation fragment, the *Synechocystis* enzymes reacts like the RNase II of *E. coli*

**Figure 6:**
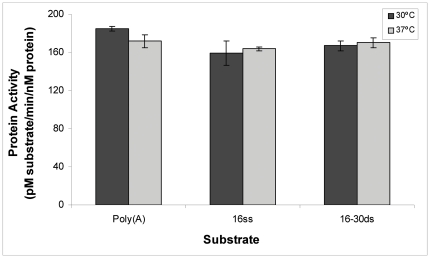
Determination of the activity of synRNB at two different temperatures. The activity of the protein was determined at 30°C and 37°C using three different synthetic substrates: 30-mer poly(A), 16-mer and the double-stranded 16–30 ds. All the activity assays were performed in triplicate.

**Figure 7:**
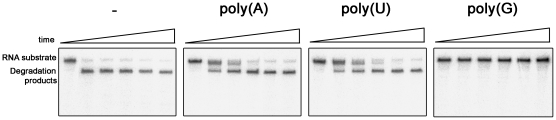
Competitive inhibition assays of synRNB by poly(A), poly(U) and poly(G) substrates. 25 nM of protein was incubated with 22 ng/µl of radiolabeled *petD3′* RNA. Unlabelled competitors were added in a 7-fold molar excess over the labelled substrate.

We then asked if the synRNB degrades poly(A) better than randomized sequenced RNA. To this end, we compared the activity of the *Synechocystis* protein with poly(A) and 16 ss substrates. The results showed that the protein had similar activities for both substrates (Figure 6). This indicates that, in contrast to the *E. coli* RNase II, which prefers poly(A) substrates [15], [17], the synRNB does not have such preference. Interestingly, while in *E. coli* the polyadenylation is performed mainly by PAP (which generates homopolymeric tails), and to some extent also by PNPase, in cyanobacteria those tails are exclusively synthesized by PNPase and are heteropolymeric [1]. Therefore, the substrate preference of members of the RNase II family may reflect the composition of 3′ tails in their organism of origin. To further examine the preference for 3′ poly(A) tails, we performed a competitive degradation assay. In this experiment, *petD3* RNA was incubated with synRNB in the presence of poly(N) oligomers. The aim was to verify if the addition of unlabelled poly(N) oligomers would compete for *petD3* degradation. The results confirmed that, in fact, poly(A) is not a preferred substrate for synRNB, since the inhibition of the degradation by both poly(A) and poly(U) was similar. However, poly(G) was able to strongly inhibit the degradation activity (Figure 7). These results confirmed that synRNB does not prefer poly(A) substrates and had higher preference for poly(G), in contrast to what was shown for the *E. coli* counterparts [15], [17]. In fact, for *E. coli* RNase R, poly(G) was practically inactive as substrate [28]. Also, the activity of both *E. coli* RNase II and RNase R is higher for poly(A), then poly(U) and finally poly(C) [28], [29].

To verify that the binding affinity of the enzyme to poly(A) and other RNA is similar, we determined the dissociation constants by SPR using different single-stranded substrates. One of the substrates was constituted only by adenosines and the second with a random sequence. The results presented in Table 1 showed that the *K*_D_ values obtained for synRNase II with both single-stranded substrates are similar (3.9±0.3 nM for the poly(A) and 3.3±0.5 nM for the 25 ss), and the same was observed regarding the association (*k*_a_) and dissociation (*k*_d_) rates. In contrast, the *K*_D_ values previously obtained for *E. coli* RNase II and RNase R showed that these proteins have an increased affinity for poly(A) substrates when compared to a random sequence [13], [15], [17].

**Table 1:** Kinetic parameters of RNase II-like protein from *Synechocystis sp*.

	k_a_ (1/Ms)	k_d_ (1/s)	K_D_ (nM)
**PolyA**	3,0±0,4 E03	1,9±0,1 E-05	3,9±0,3
**25 ss**	3,7±0,4 E03	1,1±0,1 E-05	3,3±0,5
**16–25 ds**	2,4±0,2 E05	1,4±0,2 E-04	2,1±0,1

The kinetic constants were determined by Surface Plasmon Resonance using Biacore 2000 with a 25-nt RNA oligomer (5′-Biotin-CCCGACACCAACCACUAAAAAAAAA-3′) and 30 nts PolyA as substrates.

The activity results presented above were obtained at 37°C, which is the optimal activity for the *E. coli* enzymes. However, the *Synechocystis* PCC6803 cyanobacteria lives in freshwater and its optimal temperature for growth is around the 30°C. For this reason, we compared the activity of synRNB with the synthetic oligomers at 30°C and 37°C. As shown in Figure 6, the specific activity of the protein is very similar at both temperatures with all substrates tested. However, the degradation of the poly(A) substrate is different at 30°C (Figure S1). While at 37°C we needed 250 nM of protein to reach the end-product of 4 nt, at 30°C we were able to detect the 4 nt fragment with 50 nM of protein (Figure 5 and S1). Moreover, while at 37°C we can observe the presence of high amounts of the intermediary fragment of 6 nt in the degradation of the 16-mer substrate, similarly to what is observed for RNase II (Figure 5), in the same conditions but at 30°C the 6 nt fragment is no longer visible after 20 min of reaction (Figure S1). These results indicate that the synRNB may have a higher affinity for small fragments at 30°C, which can explain why it is able to reach the final product more rapidly when compared to the activity at 37°C.

**Figure 8:**
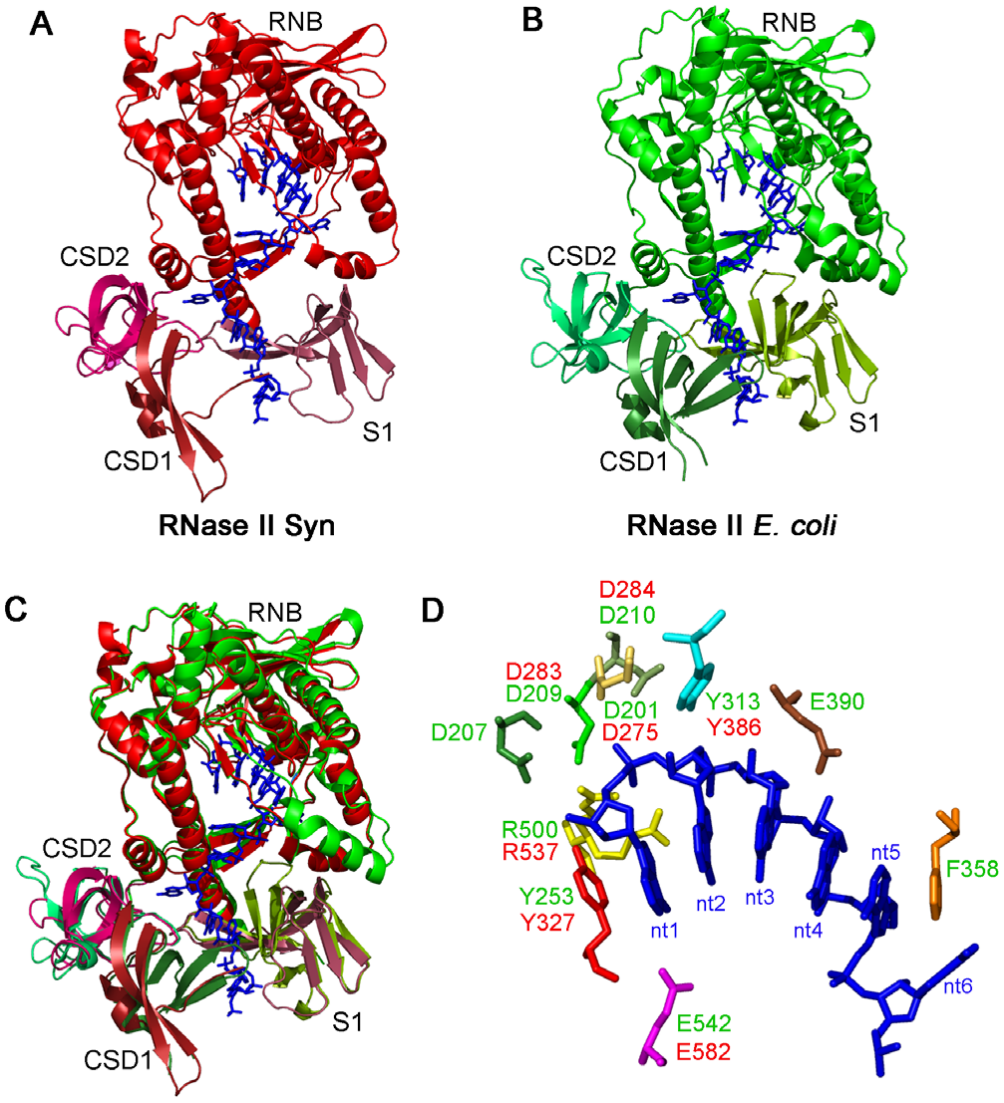
Modelling the RNase II protein from *Synechocystis*. (**A**) Representation of the predictive 3D model from *Synechocystis* RNase II (red) (B) and *E. coli* RNase II crystal structure (green) (PDB 2IX0 and 2IX1), with the RNA molecule inside (blue). (C) Superposition of *E. coli* RNase II structure and *Synechocystis* RNase II model. (D) In the catalytic cavity, the residues important for the activity of *E. coli* RNase II are shown in green, while the ones from *Synechocystis* protein are indicated in red.

We also tested the activity of the protein against the synthetic double-stranded substrate, 16–30 ds. *E. coli* RNase II is sensitive to secondary structures, and is not able to degrade this RNA, stalling 7 nt before it reaches the double-stranded region, releasing a 23 nt fragment (Figure 5) [30], [31]. In contrast, *E. coli* RNase R is able to overcome the secondary structures, releasing the typical 2 nt fragments (Figure 5) [28]. When we tested the synRNB we could observe that it was not able to overcome secondary structures. It stalled 4 nt before it reached the double-stranded region, releasing a fragment of 20 nt, which is shorter when compared to the 23 nt fragment released by *E. coli* RNase II (Figure 5). The resolution of the crystal structure from *E. coli* RNase II showed us that the catalytic cavity of the protein is only accessible to single stranded RNA due to the steric hindrance at its entrance caused by the RNA-binding domains [11]. The synRNB showed to be able to move closer to the double-stranded junction when compared to the *E. coli* protein, since that the final product released is shorter (20 nt vs. 23 nt, respectively). This may indicate that the RNA-binding domains may have a different rearrangement in this protein. In order to address this question, we modelled synRNB and compared it with *E. coli* RNase II 3D structure (Figure 8). Both proteins seemed to have a similar overall structure arrangement, with the important residues for catalysis located in the same spatial position (Figure 8D). The active site of RNase II is composed by four highly conserved aspartates and an arginine, which are important for catalysis [13], [15], [17]. Tyrosine is a residue responsible for setting the final product in the RNases from this family [13], [15], [17] (Figure 8D). In synRNB, these residues can also be found and are located in an equivalent position (Figure 8D). If we look closer to the RNA binding domains, it is possible to see that the CSD1 of synRNB (Figure 8A) is quite different from the one from *E. coli* RNase II (Figure 8B). When we superposed both structures, that difference is more noticeable (Figure 8C). Moreover, the superposition of both structures also showed that the S1 domain from SynRNB (red) lacks at least two β-sheets when compared to the one from RNase II (green) (Figure 8C). Therefore, the CSD1 from *Synechocystis* protein is more distant from the S1 domain, which could result in a wider anchoring region which in turn might allow the substrate to move nearer to the catalytic cavity, explaining why this protein is able to get closer to the double-stranded junction (Figure 8C). The activity of this protein was also determined with the 16–30 ds at both temperatures, 30°C and 37°C. Similarly to what was observed to the single-stranded substrates, the activity of the protein for the 16–30 ds is the same as observed for the other substrates and does not change with temperature (Figure 6). At 30°C, the degradation pattern of the protein remained unaltered, and the protein was still not able to overcome secondary structures, releasing a final product of 20 nt similarly to what was observed at 37°C (Figures 5 and S1). It is known that, at lower temperatures, the RNA molecules form more stable secondary structures. In *Synechocystis* PCC6803, low temperatures highly induce the expression of an RNA helicase, CrhR [32], which may be involved in the degradation of the transcripts at these temperatures by helping to unwind the secondary structures. In a strain defective in this helicase, the PNPase levels are increased up to ∼2-fold. This would help to eliminate the transcripts with cold-induced excessive secondary structures [33]. No changes were observed for synRNB protein. Together with the results described here, these findings indicate that this protein may not be involved in the degradation of double-stranded substrates at environmental temperatures. Moreover, when we determined the dissociation constants for this substrate, the value is very similar to the ones obtained for the single-stranded substrates (Table 1). However, the protein associates and dissociates more rapidly to the double-stranded substrate when compared to the other two (Table 1, *k*_a_ and *k*_d_ values).

**Figure 9:**
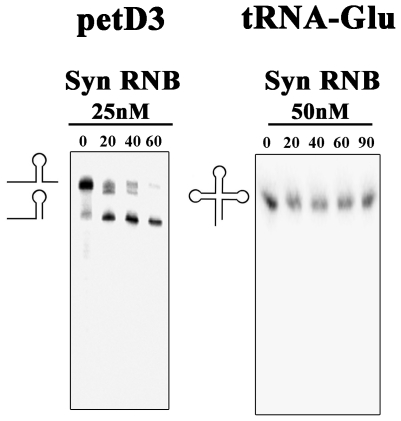
Exoribonucleolytic activity of *Synechocystis* protein with structured substrates. Activity assays were performed at 37°C as described in Materials and Methods using petD3 and tRNA-Glu as substrates. The concentration of protein used is referred. Samples were taken during the reaction at the time points indicated.

Considering that we were using a synthetic substrate, we also decided to test the activity of the recombinant protein using an mRNA and a tRNA as substrates (Figure 9). The results obtained with these substrates confirm the ones that we obtained with the synthetic 16–30 ds substrate (Figure 5). For the *petD3* RNA, which has a stem loop structure near the 3′ end, synRNB protein was only able to cleave a few nucleotides, stalling when approached the stem loop region (Figure 9). When the substrate used was the tRNA-Glu, which is a highly structured RNA molecule, the protein was not able to cleave it (Figure 9). Together, these results showed us that the *Synechocystis* homologue behaved like an RNase II protein, since that the final product released was a 4 nt fragment for the single-stranded substrates and that the protein was shown to be sensitive to double-stranded substrates (Figures 5 and 9). However, in contrast to what happens with *E. coli* RNB family members, in *Synechocystis* RNase II does not prefer poly(A) substrates. This may be related to the fact that there is no polyadenylation by a PAP enzyme and the tails are synthesized by PNPase and are heteropolymeric [1]. This protein is the only member of the RNB-family present in *Synechocystis*. To date, when only a member of this family is described in an organism, it was shown to behave like RNase R. This was the case of *Mycoplasma genitalium* [*34*], *Legionella pneumophila* [*19*], and *Streptococcus pneumoniae* [35]. *Synechocystis sp* is, the first organism where such observation was now shown not to be the case. In order to clarify why *Synechocystis* evolved to have an RNase II and not an RNase R, we decided to perform a phylogenetic analysis.

**Figure 10:**
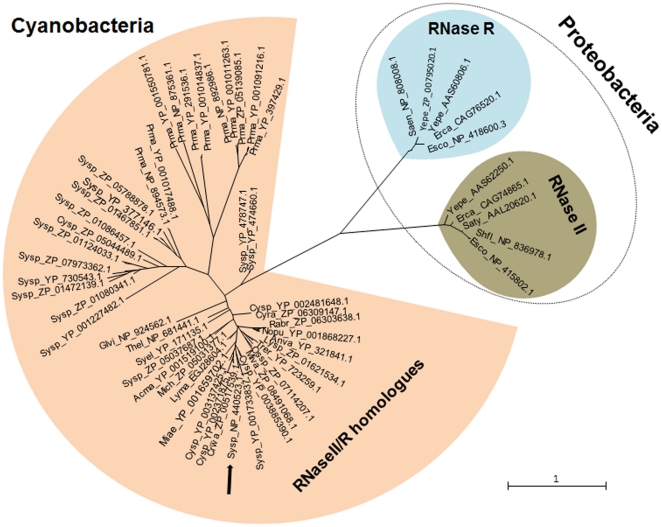
Phylogenetic relationships between 47 exoribonucleases from Cyanobacteria and 5 representative Proteobacteria members of each RNase II and RNase R families. The phylogenetic tree was constructed based on the result of the global alignment of the 57 exoribonuclease sequences using maximum likelihood at the Phylogeny.fr pipeline (http://www.phylogeny.fr/) (28). A branch length of one substitution/site is given to infer phylogenetic distances. The position of the RNase II/R homologue from *Synechocystis* sp. PCC6803 in the tree is highlighted with an arrow. Sequences are identified by the following criteria: species are represented by the first two letters of the genus followed by the first two letters of the species name; this is followed by the representative identification code issued from the GenBank database.

To analyze the phylogenetic relationship between cyanobacteria RNase II/R homologues and defined proteobacteria RNase II and RNase R enzymes, we first created a multiple sequence alignment with MUSCLE [36]. Amino acid identities between cyanobacteria RNase II/R homologues and RNase II or RNase R enzymes are restricted to the central RNB catalytic domain of these proteins. The unrooted phylogenetic tree prepared with the 57 RNase II/R homologues reveals the existence of three clusters with different evolutionary lineages, consisting of the cyanobacteria RNase II/R homologous and the proteobacteria RNase II and RNase R groups (Figure 10). Interestingly, the cyanobacterial RNase II/R group is subdivided into three subclusters indicating considerable diversity between the species. It may represent an ancestral condition for the phylum with a subsequent convergence into two specialized family of exoribonucleases, namely RNase II and RNase R (Figure 10). Moreover, the phylogenetic analysis revealed that the RNase II and RNase R members are approximately equally distant from the RNase II/R homologue present in *Synechocystis* sp. PCC6803 (Figure 10). In this work, we proved that biochemically the RNase from *Synechocystis* sp. PCC6803 behaves as an RNase II and not like RNase R, although it is equally distant from both. It is possible that the ancestor would have both homologues and one of the enzymes was lost during evolution (some organisms maintained only an RNase R-like member, while for others, like *Synechocystis*, it was more propitious and favourable to maintain the RNase II-like protein). Other hypothesis was the presence of a unique enzyme, which evolved according to the environmental conditions.

## Materials and Methods

### Overexpression and purification of recombinant RNase II from *Synechocystis sp.*

The plasmid used for expression of *Synechocystis sp.* PCC6803 histidine-tagged RNase II protein was pQE31synRNB. The *rnb* gene of *Synechocystis sp.* was amplified by PCR from genomic DNA using the primers synrnb1 (5′-GGCGAATTCATGGAAAAAGGACAACTAAT-3′) and synrnb2 (5′-GGCAGATCTAGGCYYCATTGGCCAACA-3′). This fragment was then digested and ligated into a pQE31 expression vector using *Pst*I and *Sph*I restriction sites. This allows the expression of the (His)_6_-tagged Syn RNase II fusion protein.

The plasmid was transformed into *E. coli* M15 (REP4) strain to allow the expression of the recombinant protein. Cells were grown at 37°C in 100 ml LB medium supplemented with 150 µg/ml ampicillin to an OD_600_ of 0.5 and induced by addition of 0.5 mM IPTG for 2 h. Cells were pelleted by centrifugation and stored at −80°C. *E. coli* RNase II and RNase R overexpression were performed as described previously [16], [26], [27].

Purification was performed by histidine affinity chromatography using HiTrap Chelating HP columns (GE Healthcare) and AKTA FPLC system (GE Healthcare) following the protocol described previously [16], [27]. The fractions containing the purified *Synechocystis* protein were pooled and loaded to an anion exchange monoQ column (GE Healthcare) equilibrated in buffer B composed by 20 mM Tris pH 8, 60 mM KCl, 2 mM MgCl_2_ and 0.2 mM EDTA. Protein elution was achieved by a continuous KCl gradient (from 60 mM to 1 M) in buffer B. Protein concentration was determined by spectrophotometry by using the ND100 Spectrophotometer from Nanodrop and 50% (v/v) glycerol was added to the final fractions prior storage at −20°C. 0.5 µg of the purified protein was applied to 8% SDS-PAGE and visualized by Coomassie blue staining (data not shown).

### Exoribonucleolytic activity assays

The exoribonucleolytic activity was determined using different synthetic substrates: a poly(A) oligomer of 30 nts, a 16-mer oligoribonucleotide (5′-CCCGACACCAACCACU-3′), and a 30-mer oligoribonucleotide (5′-CCCGACACCAACCACUAAAAAAAAAAAAAA-3′). The 30-mer was hybridized to the complementary unlabelled 16-mer oligodeoxiribonucleotide (5′-AGTGGTTGGTGTCGGG-3′), in order to obtain the double-stranded substrate 16–30 ds. The hybridization was performed in a 1∶1 (mol∶mol) ratio by 5 min incubation at 100°C followed by 45 min at 37°C. These RNA molecules were labelled at 5′-end with [γ-^32^ATP] and T4 polynucleotide kinase. The RNA oligomers were then purified using Microcon YM-3 Centrifugal Filter Devices (Millipore) to remove the unincorporated nucleotides. *In vitro* transcribed substrates were also used for the degradation assays. *petD3* and tRNA-Glu were transcribed from pBlueScript KS-psbA3′ [37] or from a PCR product of the coding sequence of tRNA-glu [25] using T7 RNA polymerase RIBOMAX kit from Promega (following the instructions given by manufacturers) in a 20 µl volume, containing 20 µCi of [α-^32^P] UTP. Radioactively labelled RNA transcripts were purified on a 6% PAA/7M urea gel as previously described [38].

The exoribonucleolytic reactions were carried out in a final volume of 12.5 µl containing 5 nM of substrate, 20 mM Tris-HCl (pH tested from 6.5 to 9), KCl or NaCl (from 10 to 200 mM), MgCl_2_ (from 1 to 10 mM), and 1 mM DTT. The amount of each enzyme added to the reaction was adjusted to obtain linear conditions and is indicated in the figures and respective legends. Reactions were started by the addition of the enzyme and incubated at 30°C or 37°C. Samples were withdrawn at the time points indicated and the reaction was stopped by adding formamide-containing dye supplemented with 10 mM EDTA. Reaction products were resolved in a 20% polyacrylamide/7 M urea, or TLC chromatography [25] and detected by using the Fuji Phosphorimager Analyzer TLA-5100 from GE Healthcare. The exoribonucleolytic activity of the enzyme was determined by measuring and quantifying the disappearance of the substrate in several distinct experiments. In the quantifications, the protein concentration was adjusted and less than 25% of substrate was degraded. Each value obtained represents the mean for these independent assays.

### Surface plasmon resonance analysis - BIACORE

Biacore SA chips were obtained from Biacore Inc. (GE Healthcare). The Flow cells of the SA streptavidin sensor chip were coated with a low concentration of the following substrates. On flow cell 1, no substrate was added so this cell could be used as the control blank cell. On flow cell 2, a 5′ biotinylated 25-nucleotide RNA oligomer (5′-CCCGACACCAACCACUAAAAAAAAA-3′) was added to allow the study of the protein interaction with a single-stranded RNA molecule. On flow cell 3, a 5′ biotinylated 30-mer PolyA substrate. On flow cell 4, the biotinylated 25-mer hybridized with the complementary 16-mer oligodeoxiribonucleotide (5′-AGTGGTTGGTGTCGGG-3′) was immobilized, originating the double-stranded substrate 16–25 ds. The target substrates were captured on flow cells 2 to 4 by manually injecting 20 µl of a 500 nM solution of the substrates in the reaction buffer at a 20 µl/min flow rate. The biosensor assay was run at 4°C in the buffer with 20 mM Tris-HCl pH7.5, 50 mM KCl, 1 mM DTT and 25 mM EDTA. The proteins were injected over flow cells 1, 2, 3 and 4 for 2.5 min at concentrations of 10, 20, 30, 40 and 50 nM using a flow rate of 20 µl/min. All experiments included triple injections of each protein concentration to determine the reproducibility of the signal and control injections to assess the stability of the RNA surface during the experiment. Bound protein was removed with a 30 s wash with 2 M NaCl. Data from flow cell 1 were used to correct for refractive index changes and non-specific binding. Rate and equilibrium constants were calculated using the BIA EVALUATION 3.0 software package, according to the fitting model 1∶1 Languimir Binding.

### Phylogenetic analysis

Based on sequence similarity to the *Escherichia coli* RNase II (Accession number NP_415802.1), we have identified 47 non-redundant representative homologues among cyanobacteria. Sequence similarity searches were performed using BLASTP 2.0 against cyanobacterial genomes database of the National Center for Biotechnology Information (NCBI). Next, we selected from the NCBI database five representative members from RNase II and RNase R family of enzymes, respectively. A multiple sequence alignment of the 57 RNase II/R homologues was generated by MUSCLE [36] and curated with default parameters of GBlocks [39]. Subsequently, a phylogenetic tree was constructed using the default option of the “advanced mode” implemented in the Phylogeny.fr platform (http://www.phylogeny.fr/) [40]. A bootstrap analysis of 500 replicates was used to provide confidence of the constructed tree.

### Protein modelling

The model structures were built using the Swiss Model web server (http://swissmodel.expasy.org/ [41]–[43]). 3D model for the synRNB protein was based on the crystal structures of wild-type RNase II and the RNase II D209N mutant complexed with a 13-nucleotide poly(A) RNA (Protein Data Bank entries 2IX1 and 2IX0 [11]). Figures of the structure and models were generated with Pymol [44].

## Supporting Information

Figure S1 **Exoribonucleolytic activity at 30°C of** ***Synechocystis*** **protein: comparison with** ***E. coli*** **RNase II and RNase R.** Activity assays were performed using the three synthetic substrates: 30-mer poly(A), the 16-mer and the double-stranded substrate 16–30 ds. The concentration of proteins used is indicated in the figure. Samples were taken during the reaction at the time points indicated. Control reactions with no enzyme added (*Ctrl*) were incubated at the maximum reaction time for each protein. Length of substrates and degradation products are labelled.
(JPG)
