# Correction: The *rnb* Gene of *Synechocystis* PCC6803 Encodes a RNA Hydrolase Displaying RNase II and Not RNase R Enzymatic Properties

**DOI:** 10.1371/journal.pone.0102745

**Published:** 2014-07-08

**Authors:** The PLOS ONE Staff

In the Funding section, the project grant number from the funder Fundação para a Ciência e a Tecnologia (FCT) is missing. The correct grant number is: PTDC/AGR-GPL/100509/2008.
