# Supplementary figures and images for: The rnb Gene of Synechocystis PCC6803 Encodes a RNA Hydrolase Displaying RNase II and Not RNase R Enzymatic Properties
Source: PLoS One. 2012 Mar 5;7(3):e32690. doi: 10.1371/journal.pone.0032690 (PMC3293843; doi:10.1371/journal.pone.0032690)

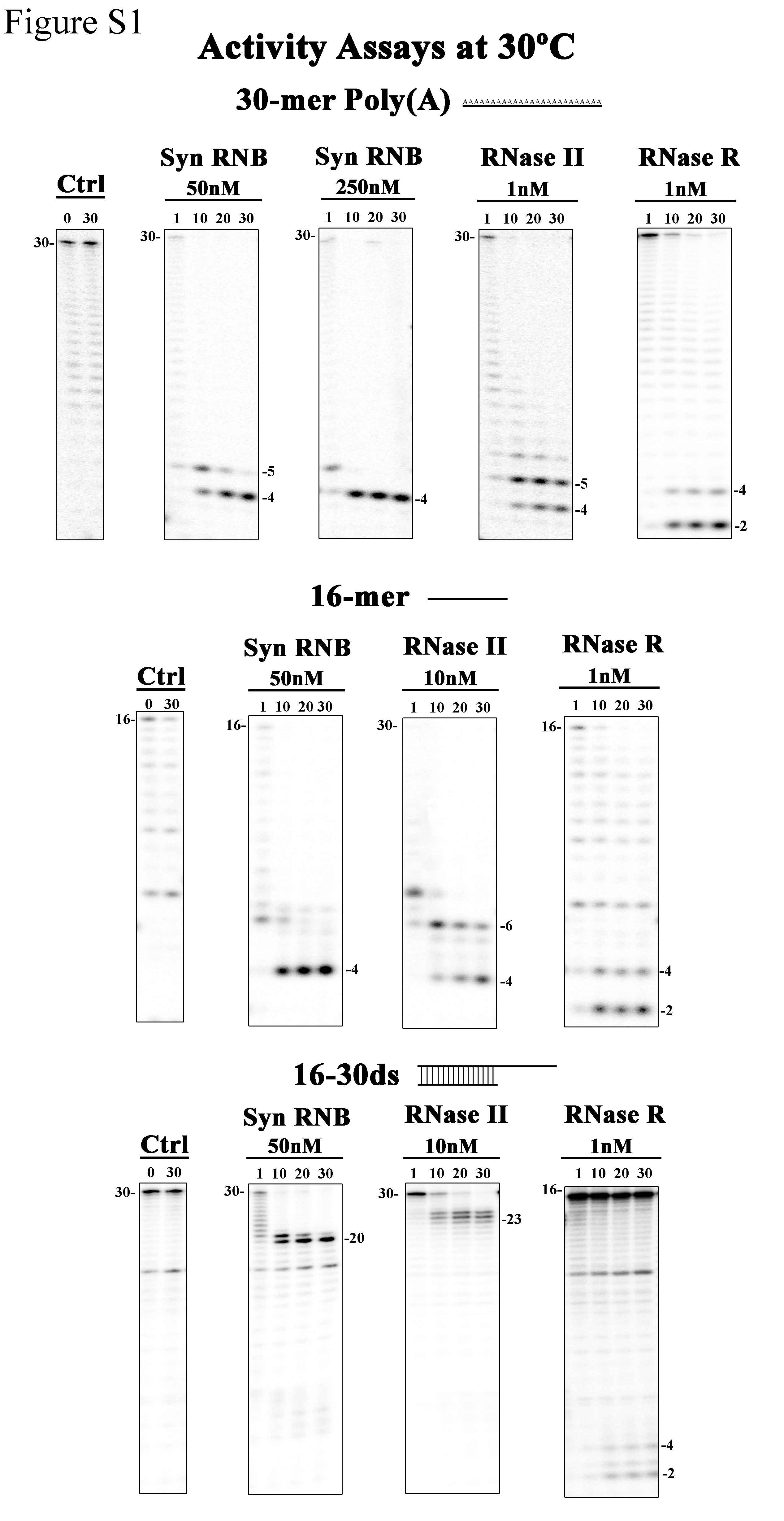

Supplement: Figure S1 — Exoribonucleolytic activity at 30°C of Synechocystis protein: comparison with E. coli RNase II and RNase R. Activity assays were performed using the three synthetic substrates: 30-mer poly(A), the 16-mer and the double-stranded substrate 16–30 ds. The concentration of proteins used is indicated in the figure. Samples were taken during the reaction at the time points indicated. Control reactions with no enzyme added (Ctrl) were incubated at the maximum reaction time for each protein. Length of substrates and degradation products are labelled. (JPG) [file pone.0032690.s001.jpg]
